# Gender-Related Differences in Chest Pain Syndromes in the Frontiers in CV Medicine Special Issue: Sex & Gender in CV Medicine

**DOI:** 10.3389/fcvm.2021.744788

**Published:** 2021-11-17

**Authors:** Puja K. Mehta, Janet Wei, Chrisandra Shufelt, Odayme Quesada, Leslee Shaw, C. Noel Bairey Merz

**Affiliations:** ^1^Division of Cardiology, Department of Medicine, Emory Clinical Cardiovascular Research Institute and Emory Women's Heart Center, Emory University School of Medicine, Atlanta, GA, United States; ^2^Barbra Streisand Women's Heart Center, Cedars-Sinai Smidt Heart Institute, Los Angeles, CA, United States; ^3^Women's Heart Center, The Christ Hospital Heart Institute, Cincinnati, OH, United States; ^4^Department of Radiology, Weill Cornell Medicine, New York, NY, United States

**Keywords:** sex, gender, chest pain, coronary artery disease, INOCA

## Abstract

Coronary artery disease (CAD) is the leading cause of morbidity and mortality among both women and men, yet women continue to have delays in diagnosis and treatment. The lack of recognition of sex-specific biological and socio-cultural gender-related differences in chest pain presentation of CAD may, in part, explain these disparities. Sex and gender differences in pain mechanisms including psychological susceptibility, the autonomic nervous system (ANS) reactivity, and visceral innervation likely contribute to chest pain differences. CAD risk scores and typical/atypical angina characterization no longer appear relevant and should not be used in women and men. Women more often have ischemia with no obstructive CAD (INOCA) and myocardial infarction, contributing to diagnostic and therapeutic equipoise. Existing knowledge demonstrates that chest pain often does not relate to obstructive CAD, suggesting a more thoughtful approach to percutaneous coronary intervention (PCI) and medical therapy for chest pain in stable obstructive CAD. Emerging knowledge regarding the central and ANS and visceral pain processing in patients with and without angina offers explanatory mechanisms for chest pain and should be investigated with interdisciplinary teams of cardiologists, neuroscientists, bio-behavioral experts, and pain specialists. Improved understanding of sex and gender differences in chest pain, including biological pathways as well as sociocultural contributions, is needed to improve clinical care in both women and men.

## Introduction

Coronary artery disease (CAD) is the leading cause of morbidity and mortality among both women and men (1, 2). However, sex disparities in CAD outcomes persist, as women are more often underdiagnosed or delayed in diagnosis (3, 4), receive less guideline-based treatment (5, 6), and are not included proportionately in clinical trials (7). Women more often have pre-hospital delay in presentation after chest pain onset (by ~30–45 min compared to men) (3, 8, 9). Compared to men, women are underdiagnosed for myocardial infarction, less likely to undergo coronary angiography, and less likely to receive therapies such as revascularization and mechanical circulatory support (8, 10–13).

**Figure 1:**
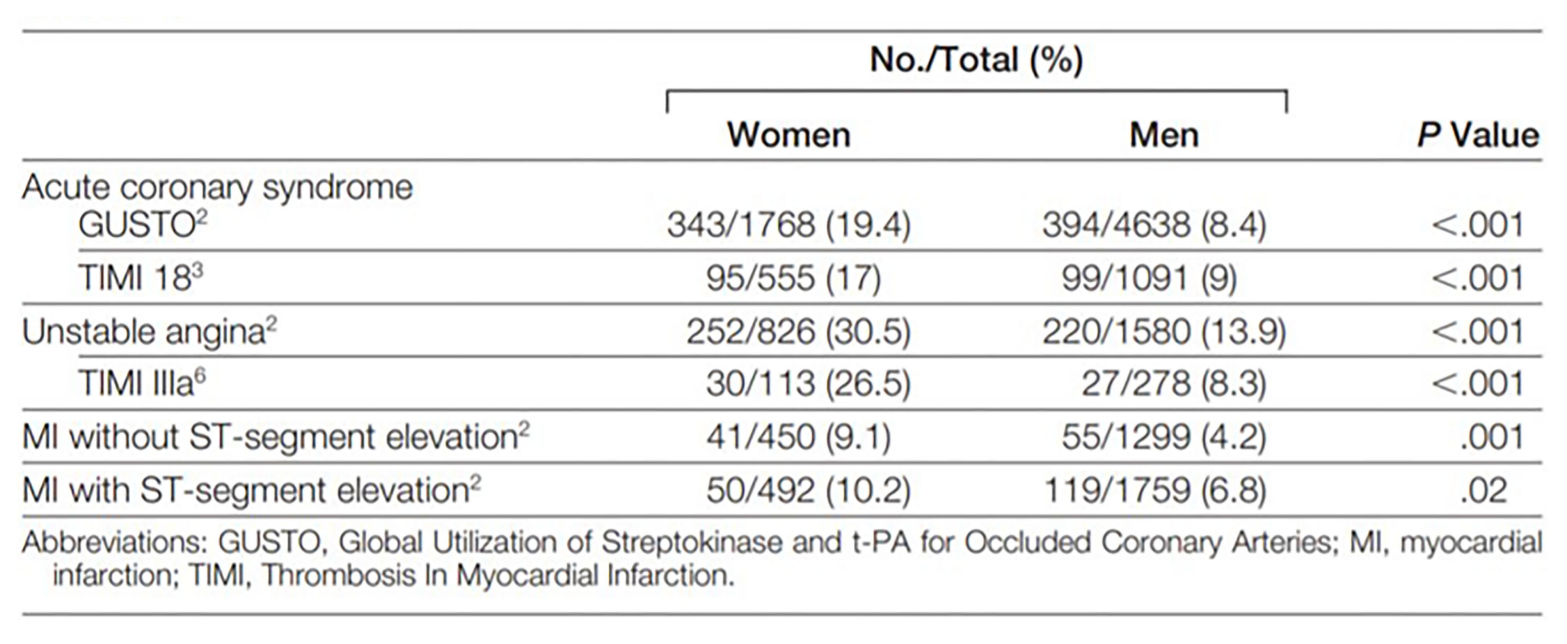
Prevalence of “Normal” and non-obstructive coronary arteries in women and men. Normal (no visible angiographic disease) or non-obstructive coronary arteries (luminal irregularities <50%) is found more often in women than men who undergo invasive coronary angiography for acute coronary syndrome and ST-segment elevation myocardial infarction. *Reprinted with permission* (14).

Excess mortality in women appear to be driven by age, as women are older with more co-morbidities which may contribute to delays in aggressive treatment, and lower preventive therapy compared to men (4, 5). In a recently published large meta-analysis of 705,098 patients with STEMI (31% women), women had higher in-hospital mortality (OR 1.91), repeat myocardial infarction (OR 1.25), stroke (OR 1.67), and major bleeding (OR 1.82) compared to men (8). Furthermore, women more often have no obstructive CAD in the setting of acute coronary syndrome (ACS), NSTEMI, and STEMI (Figure 1), (14) potentially contributing to diagnostic and therapeutic equipoise. Even though standardized STEMI protocols appear to eliminate sex-differences in age-adjusted mortality, contemporary data demonstrate persistent delayed contact-to-reperfusion time, and less guideline-recommended medical therapy in women compared to men (15–17).

## Sex and Gender Definitions

Sex is defined as a person's biological status and is usually categorized as male, female, or intersex. Biological sex is often indicated by the sex chromosomes and the gonads. Sex as a biological variable (SABV) consideration is now an important component of biomedical scientific study rigor (18). Gender is defined as socially constructed characteristics of women and men, and refers to the norms, roles, and relationships of and between groups of women and men. Gender varies from one society to another and can be changed as the socio-cultural attributes of the sex. Sex and gender are different concepts that are often used interchangeably, but within biomedical research sex is biologically defined, and gender remains a social construct relative to the individual and others perception of themselves as a man or a woman, or another gender identity.

## Chest Pain Definitions

**Figure 2:**
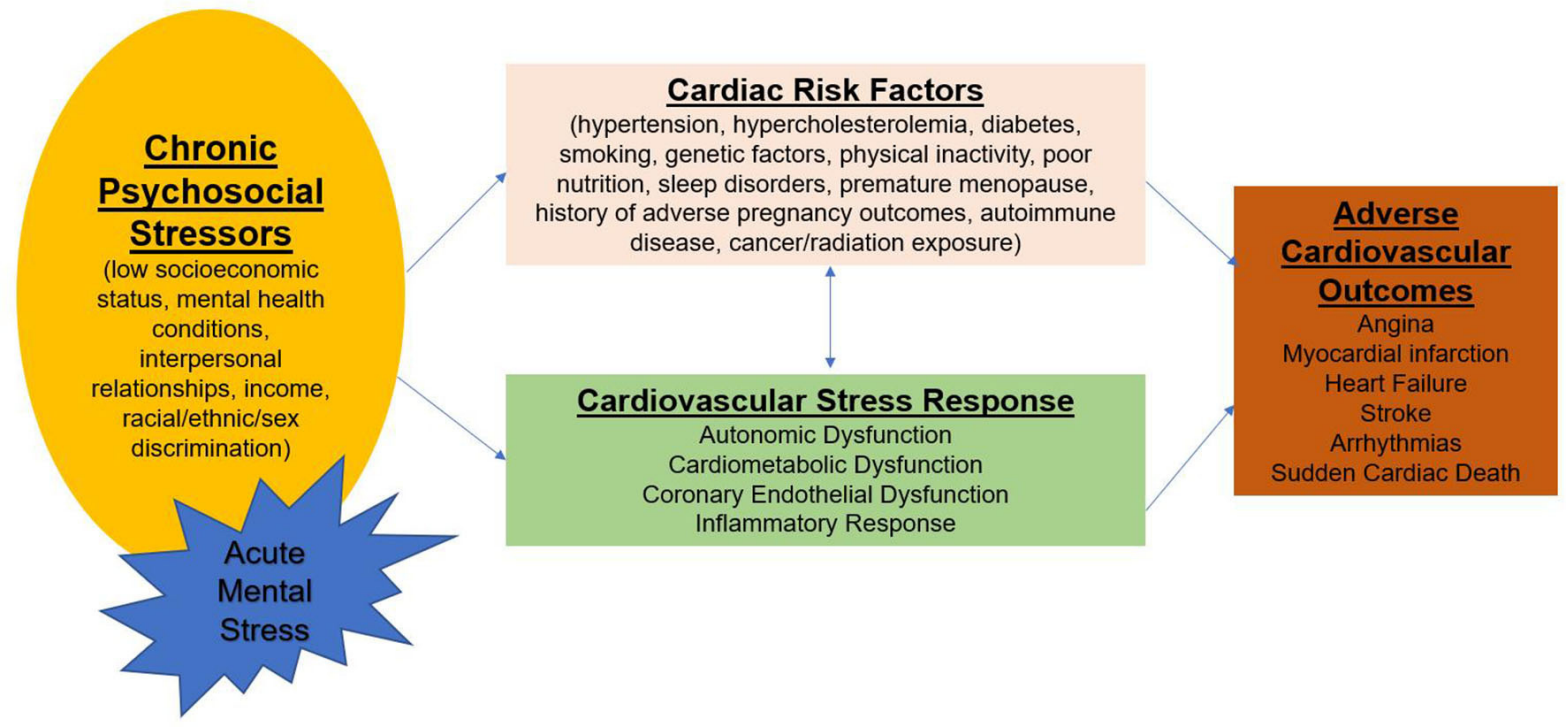
Psychological stress, risk factors, and cardiovascular disease. Acute episodes of mental stress superimposed on chronic stress influence the cardiovascular stress response involving autonomic dysfunction, cardiometabolic dysfunction, endothelial dysfunction, and inflammatory pathways. These pathways are modulated by cardiac risk factors leading to adverse cardiovascular outcomes.

Chest pain is defined as discomfort or pain occurring anywhere between the jaw and upper abdomen. Chest pain is a subjective symptom and can have a wide differential, which requires thorough history taking and often additional diagnostic testing. One of the most important etiologies is cardiovascular disease, the leading killer of women and men (1, 19). Chest pain often does not correlate with objective measures of myocardial ischemia or obstructive CAD and is influenced by psychological status (20), suggesting an inclusive approach to chest pain symptom etiology. Emerging knowledge of the cardiovascular stress response to psychosocial stressors in patients with cardiac risk factors has recently been reviewed and may provide insight into angina and other cardiovascular outcomes (Figure 2) (21). Accordingly, chest pain can have biological sex contributions as well as socio-culturally determined gender contributions.

## Sex/Gender Contributions to Chest Pain

Sex differences in pain perception are well-described, where female sex has higher somatic awareness compared to male sex (22). This potentially leads to women having greater sensitivity but lower specificity for cardiac chest pain. Further, differing phenotypes of biological sex impact pain perception, e.g., younger premenopausal women with relatively high estrogen levels have a greater pain perception compared to older postmenopausal women with lower estrogen levels (23). Younger pre-menopausal women are erroneously thought to be “protected” from CAD, and younger women's pain symptoms are more easily discounted. Specifically, socio-cultural gender is documented to contribute to subjective symptoms, where gender bias in pain diagnosis and treatment has been identified within the patient-provider encounter and treatment decisions (24). A comprehensive evaluation of sex and gender differences in pain includes proximate cause contributions of experiential (abuse, labor, and delivery), psychological (anxiety, depression, post-traumatic stress), genetic (X chromosome imprinting/Y chromosome), neurochemical (adenosine, cytokine expression), organizational (steroid action in development), activational (steroid action in adulthood), systems level (cortical connectivity, vagal nerve modulation), and sociocultural (gender roles, gender role expectations) (25).

## Sex/Gender in Chest Pain and Psychological Status

Comorbid psychological conditions such as anxiety, depression, and post-traumatic stress disorder are highly prevalent in both women and men with CAD, and psychological stress can exacerbate angina (26–29). Depression is associated with chest pain, regardless of CAD severity (27, 30), and Pimple et al. have reported that in women, but not in men, chest pain frequency was associated with more mental stress-triggered ischemia detected by nuclear imaging (31). Mental stress-triggered ischemia predicts a 2-fold higher mortality, and abnormal autonomic response to stress leading to increased coronary vascular reactivity is implicated (32, 33). We have observed greater peripheral microvascular constriction using peripheral arterial tonometry (PAT) during a mental stress test in women with INOCA compared to matched asymptomatic controls (34, 35). A greater number of INOCA subjects had chest pain during mental stress test compared to controls (41 vs. 10%, *p* = 0.01). Higher anxiety and frustration during mental stress correlated with peripheral vasoconstriction, and compared to asymptomatic controls, those with INOCA remained more anxious, frustrated, and irritated post mental stress testing (35).

## Sex/Gender in Chest Pain and the Autonomic Nervous System

A relatively large portion of women and men with objective evidence of ischemia or myocardial infarction have coronary microvascular dysfunction (CMD) despite absence of obstructive CAD (36–38). Although more women than men appear to be referred for advanced CMD evaluation, female-specific mechanisms are not well-understood (38–40). Risk factors such as hypertension, diabetes, systemic inflammation, and estrogen deficiency have all been implicated, however these factors do not predict CMD-related chest pain (41–43). Failure to auto-regulate myocardial blood flow due impaired microvascular function implicates ANS dysfunction as an important mechanism in CMD-related chest pain syndrome. While increased sympathetic activity due to stimuli such as mental stress may lead to enhanced vasoconstriction, CMD patients are a complex and a heterogeneous group, where a subset appears to have increased cardiac pain sensitivity and high somatic awareness. Prior studies have shown that compared to those with angina from obstructive CAD, patients with no obstructive CAD report more pain with contrast injection in the coronaries, with right ventricle pacing, and with adenosine infusion; furthermore, pain at a lower stimulus intensity is observed in these patients (44–47). However, it is not known whether the exaggerated pain sensitivity is due to abnormal sympathetic activation in the heart vs. abnormal ANS processing of visceral afferent signals (46, 48).

## Sex/Gender and Chest Pain and the Central Nervous System

The cortico-limbic structures play an important role in emotional regulation, pain processing, and cardiovascular sympathetic outflow (49, 50). Increased pain sensitivity appears to result from abnormal cortical processing of pain signals. Brain activation was reported in the hypothalamus, periaqueductal gray, thalami, the prefrontal cortex, and the left inferior anterior cingulate cortex (ACC) during chest pain in obstructive CAD patients with ischemia (51, 52). Conversely, patients with asymptomatic ischemia did not demonstrate increased frontal cortex activity, although thalamic activation was similar to the chest pain group (51), suggesting that abnormal visceral pain processing of afferent pain signals may be present in mental stress-triggered ischemia. In the Mental Stress Ischemia Prognosis Study (MIPS), CAD patients with angina had increased activation with mental stress in the ACC and associated regions in the inferior frontal gyrus and parietal cortex, compared to those without angina (53). The ACC is a component of the limbic circuit, has extensive connectivity to the insula, amygdala and autonomic centers, and plays a role in processing emotional and fear responses, learning, pain processing, and autonomic cardiovascular responses (54, 55). Sex-differences in brain activation patterns during emotional stimuli have been reported, particularly in the amygdala and ACC, with women demonstrating greater activation during negative emotion (56). In particular, heightened amygdalar activity appears to be related to increased risk of Takotsubo syndrome, a condition that is often triggered by emotional or physical stress and predominates in women (57).

## Sex/Gender Differences in Chest Pain and Risk Scores

**Figure 3:**
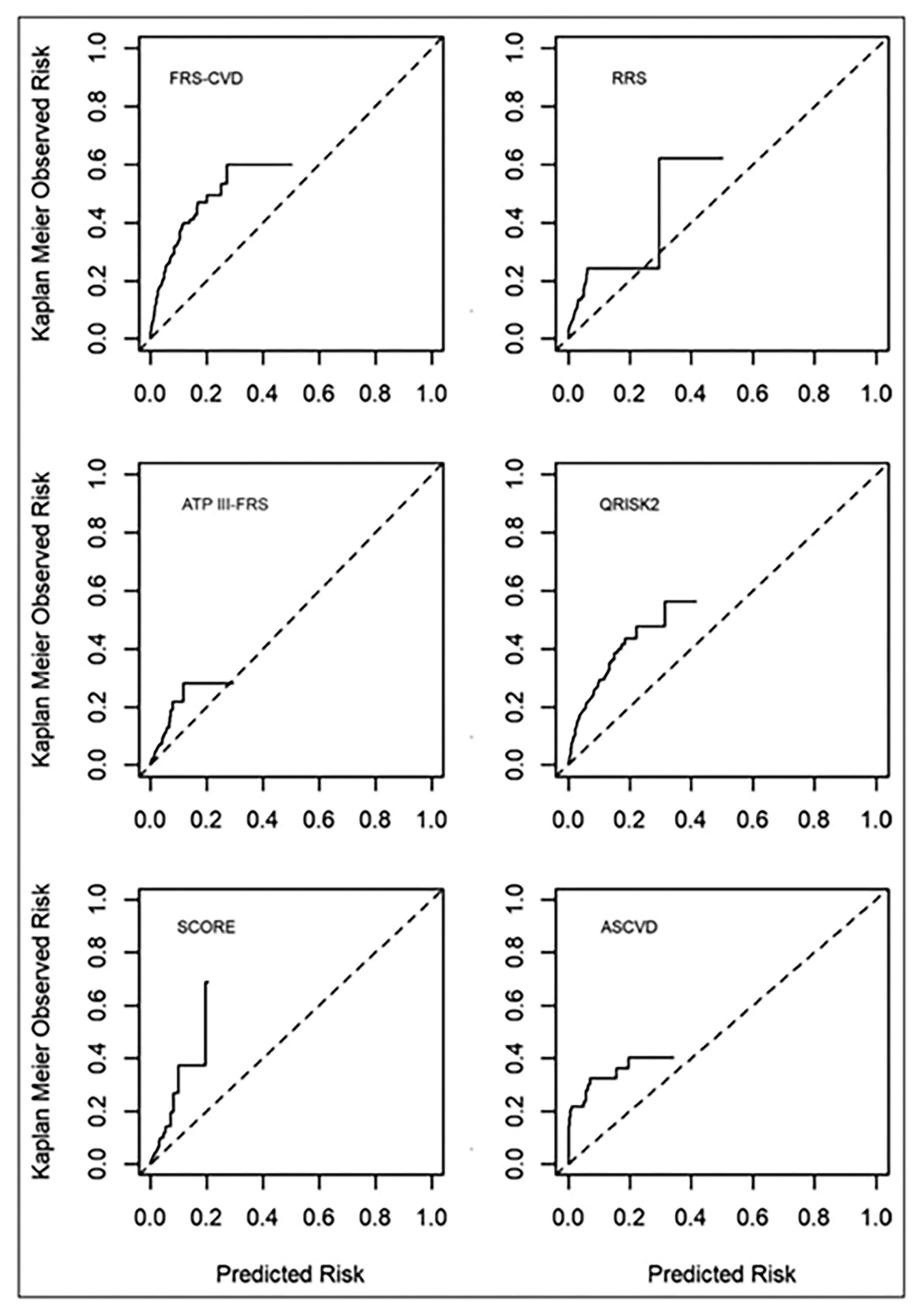
Calibration plots of risk score-specific predicted vs. observed rate of events. Commonly used cardiovascular risk scores significantly underestimate risk in women with ischemia and no obstructive CAD. Dotted line is the reference line for equal predicted and observed risk. Solid line is the observed risk. ASCVD, Atherosclerotic Cardiovascular Disease risk score; ATP III-FRS, Adult Treatment Panel III risk score; FRS, Framingham Risk Score; QRISK2, cardiovascular risk score; RRS, Reynolds Risk Score; and SCORE, Systematic Coronary Risk Evaluation. *Reprinted with permission* (66).

Several chest pain evaluation tools are available for prediction of CAD and adverse events such as myocardial infarction or cardiac death. Both acute and stable suspected CAD presentations differ between women and men (58, 59). Specifically, female sex has been shown to influence the entire diagnostic pathway for suspected CAD, from recognizing baseline risk factors to gendered referral to non-invasive testing. Sex and gender differences are well-described in questionnaire tools, including the Rose Questionnaire (60), the Diamond and Forrester tool (61), the updated Diamond-Forrester score (UDF), CAD Consortium clinical score (CAD2), and CONFIRM risk score (CRS), (62) the Duke Clinical Score (61, 63). Specifically, these tools developed predominantly in and for men have lower diagnostic accuracy for detection of acute myocardial infarction and stable obstructive CAD in women, resulting in greater “missed” myocardial infarctions (64), fewer CAD testing referrals (5), less CAD treatment and higher major adverse cardiac event rates (3) in women. Investigation aimed at developing a “female” angina tool, the Women's Ischemia Symptom Questionnaire (WISQ) did not substantially improve diagnostic value (65). Further, we have demonstrated that traditional CAD risk scores underestimate major adverse cardiovascular event rates in women with chest pain and no obstructive CAD (Figure 3) (67).

## Sex/Gender Differences in Chest Pain Characterization: Typical and Atypical Angina

**Figure 4:**
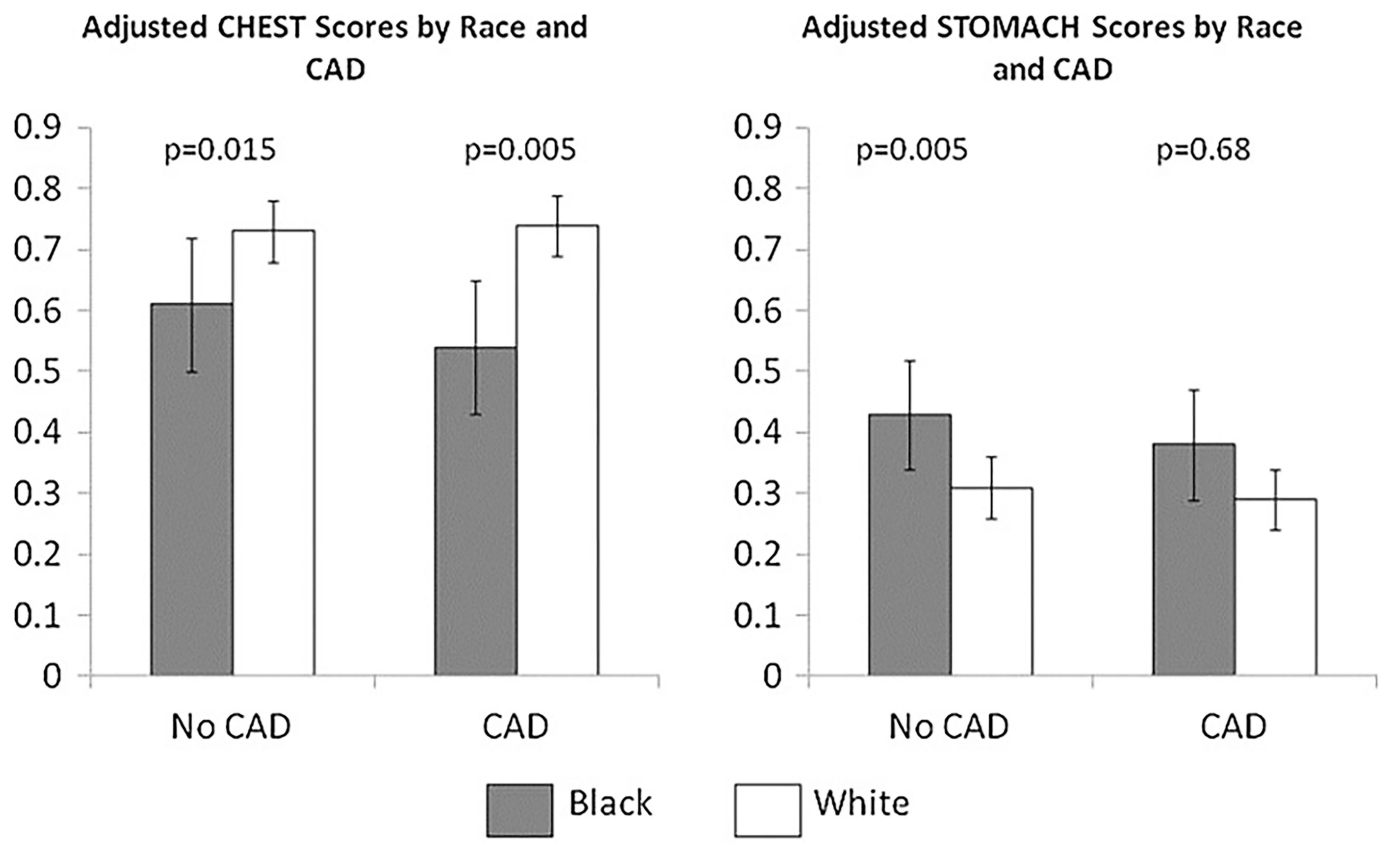
Stomach and chest angina symptoms in black women vs. white women with suspected CAD. Symptom clusters were derived from a cohort of 466 women (69 black,397 white) undergoing coronary angiography for suspected CAD. Chest symptoms included chest discomfort, pressure, tightness, fatigue, and shortness of breath. Stomach symptoms included indigestion, esophagus, throat, and abdomen symptoms. Mean adjusted chest and stomach scores stratified by race (black vs. white) and presence vs. absence of obstructive CAD. *Reprinted with permission* (76).

Prior analyses of sex and gender-based differences of angina characterization in ACS have demonstrated varied results, but the majority of studies describe chest pain as the most frequent symptom in both genders (68–70). However, women are more likely to have atypical angina, which can arise after exertion, be triggered by mental stress or even occur at rest (71). In addition, atypical angina may occur not only in the substernal region but also in the arms, jaw, neck, and upper back pain, and these atypical locations are prevalent in women with STEMI (72). Symptoms may last intermittently over several hours, and atypical symptoms may include dyspnea, unusual fatigue, dizziness, and nausea (73, 74). Contemporary cohorts demonstrate that the typicality of angina no longer discriminates obstructive CAD. Specifically, there is marked overestimation of obstructive CAD prevalence by the standard probability methods focused on the typicality of angina in both sex and age subgroups, with the most severe overestimation in women (75). Further, we have described likely socio-cultural aspects of chest pain where Black women who more often ascribe their chest pain to their stomach had a more adverse cardiovascular prognosis in this group (Figure 4) (76). These findings indicate that chest pain “typicality” should no longer guide clinical decisions, particularly in women.

## Sex/Gender in Silent Ischemia

Silent ischemia investigations from ambulatory monitoring demonstrate that more than half of “angina” episodes lack evidence of myocardial ischemia by ECG, while 85% of ambulatory ischemia (detected by ECG changes) is symptomatically “silent” (77). In obstructive CAD patients majority of chest pain episodes do not have ST depressions on ambulatory ECG monitoring (77–85). In women with CMD diagnosed by invasive coronary function testing, ambulatory ischemia is prevalent based on 24-hour ECG monitoring, but a majority of episodes are silent, and symptoms did not correlate with ST segment changes (86). Further, the severity of ischemia on stress testing does not correlate with angina burden (84, 87–89). Low heart rate variability is associated with myocardial ischemia, implicating cardiac ANS in the pathophysiology of ischemia (90, 91).

## Sex/Gender in Chest Pain and Ischemic Heart Disease

Women with evidence of ischemia, identified by objective evidence such as abnormal stress or biomarker testing, are more likely to present with no obstructive coronary arteries (INOCA) compared to men, although women report more chest pain. Traditionally, the presence of ischemic ECG changes in the absence of wall motion abnormalities on stress echocardiography is labeled as a “false-positive” stress test, but emerging data indicate that abnormal stress ECG regardless of echocardiographic response should be considered prognostic (92–94). Additional investigations should be considered to evaluate CMD and vasospasm, as ischemic ECG changes have high specificity for CMD in patients with non-obstructive CAD (95) and a high prevalence of coronary vasospasm is found in patients with typical exertional angina (96).

**Figure 5:**
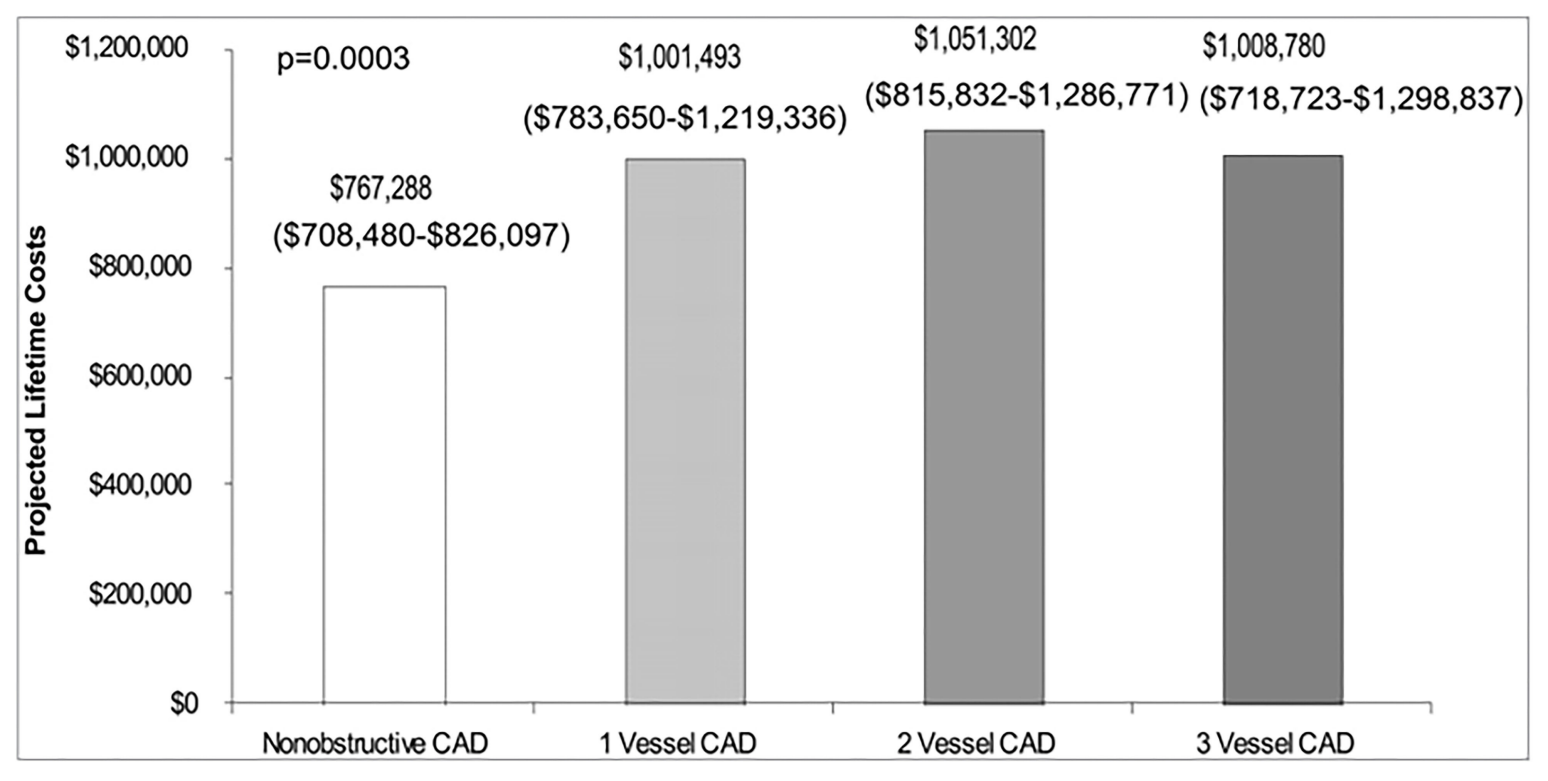
The economic burden of angina in women with suspected ischemic heart disease. Estimated lifetime costs (including sensitivity analyses ranges) of pharmacologic therapy and hospitalization for cardiovascular disease in women with non-obstructive and 1-vessel to 3-vessel CAD. Reprinted with permission (103).

**Figure 6:**
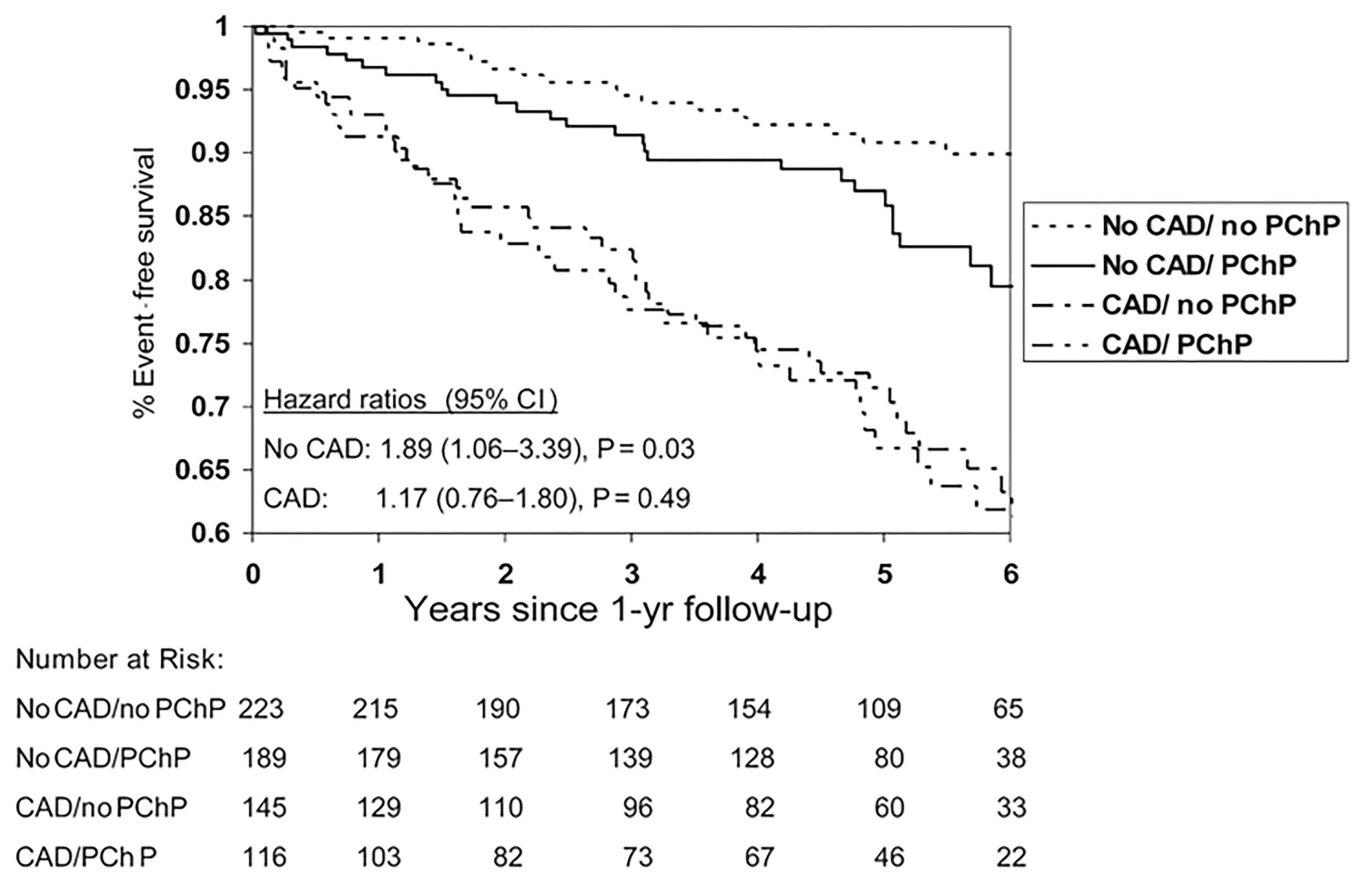
Event-free survival from cardiovascular events by coronary artery disease and persistent chest pain. Cardiovascular events for women with and without persistent chest pain (PChP) in subgroups with and without obstructive CAD. Cardiovascular events defined as cardiovascular death, MI, CHF, or stroke. *Reprinted with permission* (105).

Among patients with no obstructive CAD, endothelial dysfunction is independently associated with ischemia on stress imaging, but not with symptoms (97). Similarly, impaired coronary flow reserve is often detected in women with angina but no obstructive CAD using various invasive and non-invasive modalities (36, 38, 98, 99), but flow reserve has not been found to be associated with angina burden in women (100). However, women with angina and CMD have reduced exercise capacity compared to asymptomatic women (101). The gap between angina and identifiable ischemia on stress testing has contributed to women without obstructive CAD being diagnosed with non-cardiac chest pain, and discharged from subspecialty care and treatment (102). We have demonstrated chest pain hospitalization rates continue at a relatively constant rate in INOCA women despite medical advances (Figure 5) (104), suggesting inertia in this area. Notably, lifetime healthcare costs of chest pain in the setting of INOCA with non-obstructive CAD are close to the costs of obstructive CAD in women (Figure 6) (103).

Women are also more likely to present with myocardial infarction with non-obstructive coronary artery disease (MINOCA) (106) Recent study found that a cause of MINOCA was identified in 84.5% of the women who underwent multi-modality imaging (98/116) including optical coherence tomography (OCT) and cardiac magnetic resonance imaging (CMRI). On CMRI an ischemic pattern was present in 53.4% of participants, while a non-ischemic pattern (myocarditis, takotsubo syndrome or non-ischemic cardiomyopathy) was present in 20.7% (107) In the CIAO-ISCHEMIA (Changes in Ischemia and Angina over One year in ISCHEMIA trial screen failures with INOCA) cohort study, ischemia by stress echocardiography did not correlate with angina (108). However, in a randomized placebo-controlled clinical trial with ranolazine, change in myocardial perfusion reserve index directly correlated with change in angina measured by the Seattle Angina Questionnaire, supporting a link between symptoms and microvascular ischemia in women with INOCA (109).

Invasive coronary function testing can diagnose coronary vascular dysfunction (epicardial and microvascular) in patients with persistent angina with and without obstructive CAD (98). Guided treatment of microvascular vs. vasospastic angina has been demonstrated to improve angina outcomes (110). Even in the setting of obstructive CAD, chest pain persists up to 40% of patients post-percutaneous coronary intervention (PCI) at 1-year follow-up (111). The relationships between epicardial atherosclerotic burden and microcirculatory dysfunction needs further investigation, since it is possible that a subset of patients with persistent angina despite PCI may be experiencing CMD-related ischemia (112).

**Figure 7:**
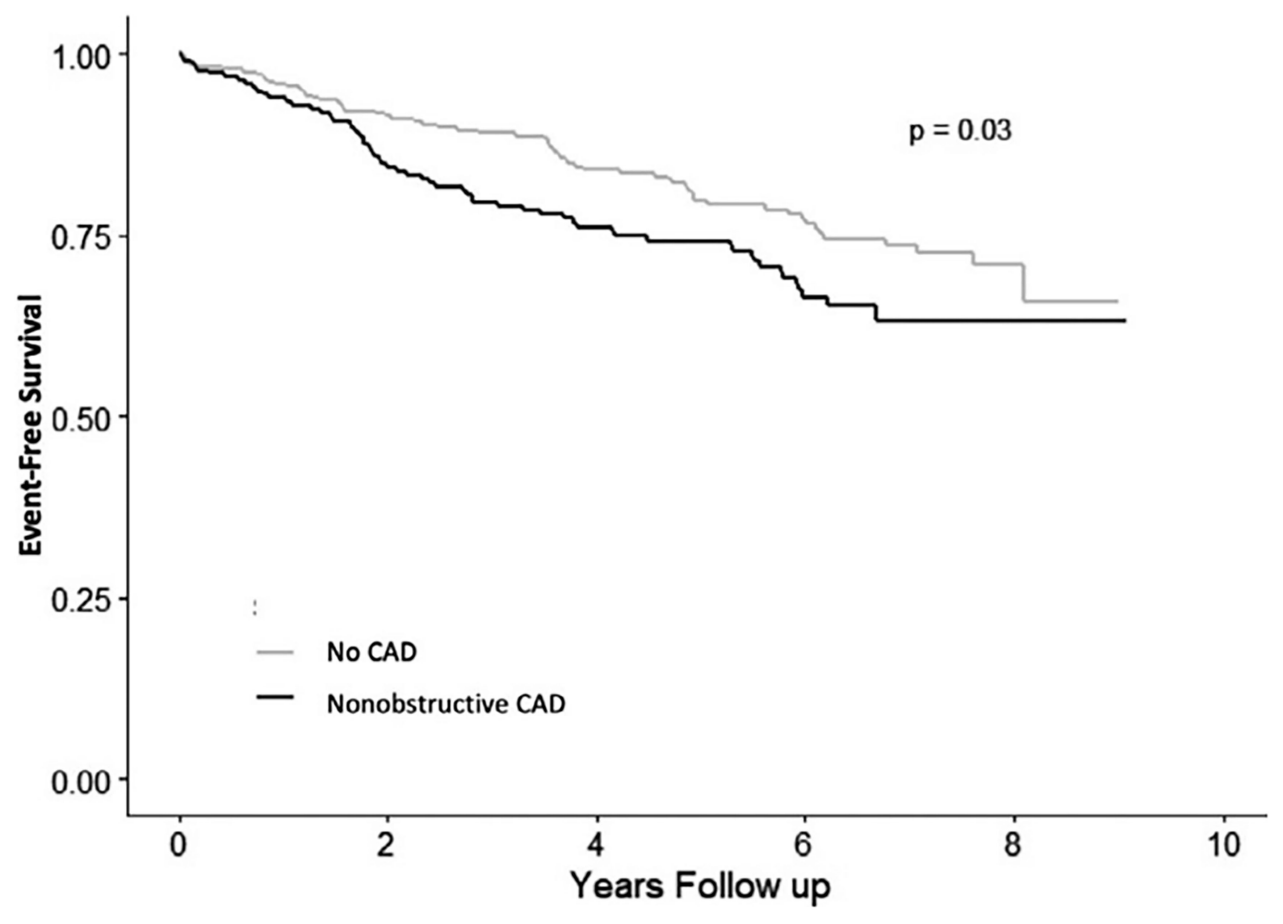
Angina hospitalization rates in women with signs and symptoms of ischemia but no obstructive CAD. Angina hospitalization event-free survival in women with no CAD (<20% stenosis) and non-obstructive CAD (≥20 to <50% stenosis). *Reprinted with permission* (104).

Recently findings from an international cohort study of patients with microvascular angina (*n* = 686, 64% women) showed that CMD is associated with significant MACE in both men and women, but women have lower quality of life compared to men (113). We have demonstrated that persistent chest pain in the absence of obstructive CAD has an adverse prognosis in women (Figure 7) (105), and atypical angina further worsens that prognosis in women (114), presumably due to a lack of recognition and treatment for underlying ischemia. Lack of recognition of sex and gender differences in chest pain may in part explain outcome disparities in women compared to men (115). CAD outcomes for women may be improved by improved provider and patient education (116, 117), standardized protocols (16), and sex-specific understanding of ischemic heart disease pathophysiology (37, 118).

## Sex and Gender Chest Pain Knowledge Gaps

Why do women have more chest pain compared to men, despite paradoxically having less obstructive CAD? Despite evidence of psychological factors contributing to angina, the brain correlates contributing to sex-differences in autonomic reactivity and persistent angina are unknown. Furthermore, whether neural mechanistic pathways can be effectively modulated to reduce angina burden and cardiovascular outcomes remains to be investigated. The biological pathways of how repetitive and cumulative chronic psychosocial stress burden over time may predispose to future angina development are unclear, especially in the context of sex differences as well as social determinants of health. The contribution of ANS activation during acute mental stress with resultant cardiovascular stress response needs to be investigated. Investigation is needed to determine whether brain activation/deactivation responses to visceral pain differ across CMD and vasospastic angina subtypes and varying degrees of CAD severity, and in comparison, to asymptomatic groups with obstructive CAD. Novel approaches that collect real-time data of daily life stress paired with autonomic output measured using wearable technology may be the next frontier to really understand psychological stress reactivity and angina. Neuro-endocrine axis disruption and inflammation as contributors to abnormal microvascular reactivity and angina in women remains to be rigorously tested. Clarifying how physiologic responses to mental stress are influenced by underlying psychological risk factors and their contribution to chest pain burden will help guide novel angina treatment strategies. Mechanistic human studies should involve interdisciplinary investigation among cardiologists, neuroscientists, bio-behavioral specialists, and pain specialists to improve chest pain understanding and treatment.

## Conclusions/Implications

The presence of persistent clinically meaningful sex and gender differences in the cardiovascular disease detection and management continues to result in cardiovascular health outcome disparities for women (119). A socio-cultural gender-bias with regard to lack of recognition of sex-differences in chest pain symptoms appear to contribute to the adverse outcome differences in women compared to men. Since chest pain often does not correlate with myocardial ischemia or obstructive CAD, a greater understanding of chest pain etiologies and CMD-specific diagnostic testing will assist with appropriate use of interventional and medical angina treatment strategies. Further investigation is needed to understand sex and gender differences in chest pain, including biological pathways as well as sociocultural contributions, to improve clinical care. Increased awareness, education, and treatment to improve the prevention of cardiovascular disease in both women and men are needed.

## Author Contributions

PM, JW, CS, OQ, LS, and CB have made a substantial, direct and intellectual contribution to the work, and approved it for publication.

## Funding

This work was supported by K23HL105787, K23HL151867, 1U54AG062334, the Edythe L. Broad Women's Heart Research Fellowship, the Constance Austin Women's Heart Research Fellowship, the Erika J. Glazer Women's Heart Research Initiative, and the Barbra Streisand Women's Cardiovascular Research and Education Program, Cedars-Sinai Medical Center, Los Angeles.

## Conflict of Interest

CB: Sanofi, Abbott Diagnostics, and iRhythm. JW: Abbott Vascular. The remaining authors declare that the research was conducted in the absence of any commercial or financial relationships that could be construed as a potential conflict of interest.

## Publisher's Note

All claims expressed in this article are solely those of the authors and do not necessarily represent those of their affiliated organizations, or those of the publisher, the editors and the reviewers. Any product that may be evaluated in this article, or claim that may be made by its manufacturer, is not guaranteed or endorsed by the publisher.
